# Acute and 6-month vascular effects of a multi-component nutritional supplement: a randomized controlled trial

**DOI:** 10.1093/ehjopen/oeag157

**Published:** 2026-11-04

**Authors:** Charles Piercy, Jenny Harris, Kenza Sackho, Paola Campagnolo, Diogo Barardo, Ben Creagh-Brown, Christian Heiss

**Affiliations:** Faculty of Health and Medical Sciences, University of Surrey, Kate Granger Building, 30 Priestley Road, Guildford GU2 7YJ, UK; Intensive Care Department, Royal Surrey County Hospital, Royal Surrey NHS Foundation Trust, Egerton Road, Guildford GU2 7XX, UK; Faculty of Health and Medical Sciences, University of Surrey, Kate Granger Building, 30 Priestley Road, Guildford GU2 7YJ, UK; Faculty of Health and Medical Sciences, University of Surrey, Kate Granger Building, 30 Priestley Road, Guildford GU2 7YJ, UK; Faculty of Health and Medical Sciences, University of Surrey, Kate Granger Building, 30 Priestley Road, Guildford GU2 7YJ, UK; NOVOS Labs, 100 Park Avenue 16th Floor, New York, NY 10017, USA; Faculty of Health and Medical Sciences, University of Surrey, Kate Granger Building, 30 Priestley Road, Guildford GU2 7YJ, UK; Intensive Care Department, Royal Surrey County Hospital, Royal Surrey NHS Foundation Trust, Egerton Road, Guildford GU2 7XX, UK; Faculty of Health and Medical Sciences, University of Surrey, Kate Granger Building, 30 Priestley Road, Guildford GU2 7YJ, UK; Vascular Department, East Surrey Hospital, Surrey and Sussex Healthcare NHS Trust, Canada Avenue, Redhill RH1 5RH, UK

**Keywords:** Flow-mediated dilation, Pulse wave velocity, Blood pressure, Dietary supplements, Vascular ageing

## Abstract

**Aims:**

To evaluate 6-month effects of a multi-ingredient nutritional powder formulation targeting complementary pathways relevant to vascular ageing on endothelial function and other vascular ageing markers in apparently healthy adults.

**Methods and results:**

The *effects of a supplement to target ageing mechanisms on vascular function study* was a double-blind, randomized, controlled trial. Participants received a multi-ingredient nutritional powder formulation comprising vitamins/minerals, amino acids, and plant-derived bioactives, or control for 6 months. Vascular assessments were performed at baseline and after 6 months (with an acute post-dose assessment). The primary outcome was change in brachial artery flow-mediated dilation (FMD). Secondary outcomes included pulse wave velocity (PWV), peripheral and central blood pressure, augmentation index, lipid profile, and estimated 10-year cardiovascular risk (SCORE2/SCORE2-OP). Sixty-one participants (baseline SCORE2/SCORE2-OP 3.6 ± 2.9%) were randomized (33 actives; 28 controls); 43 completed both visits (24 vs. 19). Compared with control, the supplement improved endothelial function (ΔΔFMD at 6 months +2.9% [95% CI 2.1–3.8]) and reduced arterial stiffness (ΔΔPWV −1.18 m/s [95% CI −2.00 to −0.36]). Peripheral systolic blood pressure (SBP) decreased (ΔΔSBP −6.1 mmHg [95% CI −10.9 to −1.3]). Lipids were unchanged. Estimated 10-year cardiovascular risk decreased (ΔΔSCORE2/SCORE2-OP −0.45% [95% CI −0.86 to −0.03]).

**Conclusion:**

Daily intake of a multi-ingredient nutritional powder formulation was associated with sustained improvements in endothelial function, arterial stiffness, and systolic blood pressure, with a modest reduction in estimated 10-year cardiovascular risk primarily attributable to lower systolic blood pressure rather than lipid change.

**Registration:**

clinicaltrials.gov NCT06145087.

## Graphical Abstract

**Graphical Abstract:**
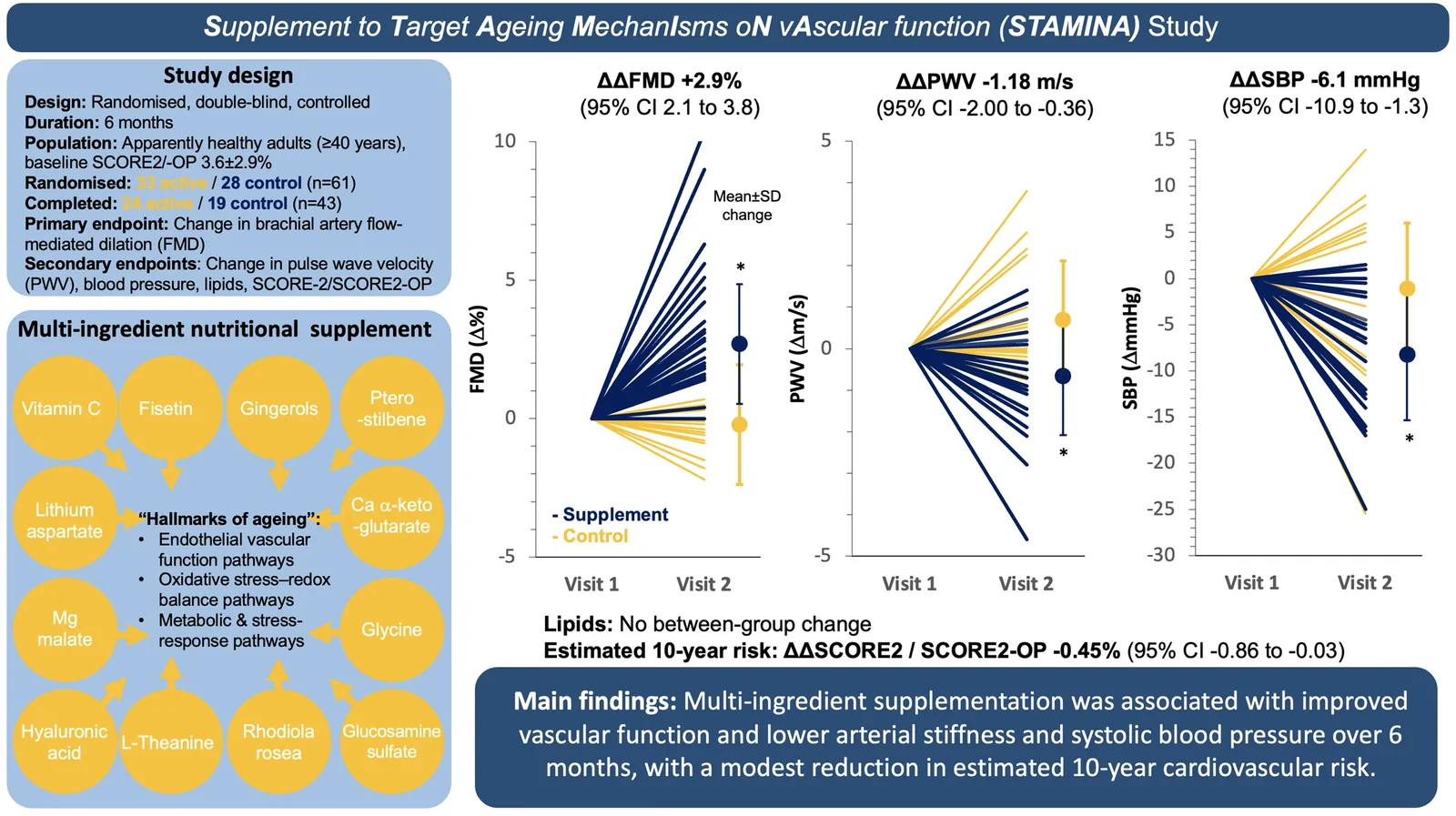

## Introduction

Vascular ageing—characterized by endothelial dysfunction, increased arterial stiffness, and raised systolic blood pressure (SBP)—is a central paradigm linking ageing to cardiovascular disease (CVD), the leading global cause of mortality.^1^ These age-associated alterations arise from interconnected biological processes including oxidative stress, low-grade inflammation, mitochondrial dysfunction, impaired nitric oxide (NO) bioavailability, and dysregulated cellular stress responses.^2^ As these mechanisms emerge long before clinical disease, interventions that improve vascular function in mid-life represent an attractive preventive strategy.^3^

A growing body of evidence suggests that specific nutritional bioactives, such as polyphenols, amino acids, and micronutrients, can beneficially modulate NO signalling, oxidative stress, autonomic balance, and endothelial health.^3–9^ While several compounds have shown efficacy individually, vascular ageing is driven by multiple converging mechanisms, raising the possibility that broader, multi-target approaches may provide additive or synergistic benefits.^3^ Because vascular ageing arises from several interacting mechanisms, single-compound interventions may only influence part of the underlying biology. A multi-component nutritional approach allows targeting several complementary pathways simultaneously while using lower doses of each ingredient, which may improve tolerability and minimize the risk of dose-related side effects. However, clinical evidence evaluating such combined strategies in humans remains limited.

We hypothesized that daily consumption of a multi-component nutritional supplement would improve endothelial function and related indices of vascular ageing compared with control. Accordingly, in this randomized, double-blind, controlled trial, we tested whether a formulation selected to combine ingredients with relevance to complementary biological pathways implicated in vascular ageing was associated with changes in endothelial function, arterial stiffness, haemodynamics, lipid profile, and estimated cardiovascular risk in healthy middle-aged and older adults (≥40 years). The primary endpoint was the change in brachial artery flow-mediated dilation (FMD) after 6 months of daily supplementation. Secondary endpoints included changes in device-derived pulse wave velocity (PWV), peripheral and central blood pressure, blood lipids, and SCORE2/SCORE2-OP-derived cardiovascular risk.

## Methods

### Trial design, participant eligibility criteria and setting

The *effects of a supplement to target ageing mechanisms on vascular function study* was a single-centre, two-armed parallel group, randomized, controlled, double-blind clinical trial with 1:1 allocation. Apparently healthy volunteers were recruited from July 2023 to January 2025 through public advertisement.

Eligible participants were aged over 40 years, self-reported as healthy, and had a BMI > 20 kg/m^2^. Exclusion criteria included any signs or symptoms of acute infection, cardiac arrhythmias, active malignancy, or unstable cardiovascular disease. Additional exclusions were applied to women who were pregnant, potentially pregnant, or had changed to hormone therapy within the preceding 2 months; individuals with psychological or traumatic conditions that could interfere with study participation; those who had received blood products or participated in another clinical trial within the previous 90 days; individuals regularly using anti-inflammatory agents, nutritional supplements overlapping with the investigational product, those with abnormal laboratory findings, and individuals with known allergies/intolerance to any of the components in the supplement product and subjects with alcohol use of more than two alcoholic beverages per day within the past month.

After assessment of eligibility, participants were enrolled and randomized to receive either a multi-component nutritional supplement (intervention group) or control. At visit 1, baseline measurements were obtained after an overnight fast. Participants then consumed their first dose under supervision, and vascular assessments were repeated 1 h post-ingestion. Participants were asked to consume treatments once daily for 6 months. Visit 2 was planned 6 months after randomization. Participants attended after an overnight fast, and vascular measurements were repeated approximately 24 h after the last daily dose. This timing was selected to avoid assessment immediately after ingestion, while still evaluating participants after repeated daily exposure to the intervention. No interim analyses or formal stopping guidelines were planned or conducted.

All researchers involved in conducting the study or assessing outcomes were masked with regard to the identity of the test products. The study protocol has received a favourable ethical opinion from the University of Surrey Ethics Committee (FHMS 21–22 237) and written informed consent was obtained from all study participants and participants received compensation for travel expenses. All volunteers were provided with a participant information sheet prior to consent and consented in the presence of a study investigator. This study was conducted in accordance with Good Clinical Practice and the Declaration of Helsinki. All patient data were pseudonymized and stored in accordance with the Data Protection Act (2018) and General Data Protection Regulation (2018). The study was registered at clinicaltrials.gov (NCT06145087).

### Interventions

The composition of the treatment intervention and control is summarized in *Table 1*. At the first visit, participants were provided with a 6-month supply of either supplement or control powder and instructed on the preparation and administration of the study supplements. Each dose of the supplement or control consisted of one level measure (15 g) using the supplied measuring spoon, dissolved in up to 500 mL of water or juice. Participants were asked to consume one full dose daily at approximately the same time each day. A dosing diary was also issued to record daily intake and monitor compliance.

**Table 1:** Composition of supplement intervention and control powder mix

	Intervention	Control
	Supplement mix	
Vitamin C (ascorbic acid)	100 mg	0
Magnesium (as magnesium malate)	304 mg	0
Calcium (as calcium alpha-ketoglutarate)	231 mg	0
Glycine	2000 mg	0
Malate (as magnesium malate)	1700 mg	0
Calcium alpha-ketoglutarate	1100 mg	0
GreenGrown® glucosamine sulphate (vegan)	1000 mg	0
*Rhodiola rosea* root extract (3% rosavins and 1% salidrosides)	300 mg	0
L-Theanine	150 mg	0
Hyaluronic acid	100 mg	0
Fisetin	100 mg	0
Organic ginger root extract (2% gingerols)	100 mg	0
Pterostilbene	50 mg	0
Lithium aspartate (providing 1 mg lithium)	20 mg	0
Other ingredients: Erythritol, malic acid, natural flavours, sodium bicarbonate, silicon dioxide, calcium silicate, and stevia	X	X

The treatments were supplied as off-white powder in identical white tubs labelled with A or B. The powders were visually indistinguishable and were matched by taste. The verum supplement (NOVOS Core, New York, USA) consisted of a multi-component formulation containing vitamins/minerals, amino acids, and plant-derived bioactives selected on the basis of prior literature suggesting relevance to biological processes implicated in ageing and vascular dysfunction. The included components were pterostilbene, glucosamine sulphate, fisetin, glycine, lithium aspartate, calcium alpha-ketoglutarate, magnesium malate, vitamin C, L-theanine, hyaluronic acid, Rhodiola rosea root extract, and ginger root extract. The control consisted of an inert matrix identical in appearance and flavouring agents without active ingredients.

### Outcomes

#### Primary endpoint: flow-mediated dilation

FMD was measured in the right brachial artery using 5 min forearm occlusion with a fitted cuff by ultrasound (Vivid 9, 12 MHz linear array, GE Healthcare, Chalfont St Giles, UK) in combination with a digital video frame grabber and semi-automated analysis system (Brachial Analyzer, MIA, Iowa City, IA, USA) as previously described.^10^ Briefly, the brachial artery was scanned proximal to the elbow longitudinally, to obtain a clear anterior and posterior vessel wall–lumen interface. In ultrasound duplex mode, the Doppler spectrum showing blood flow velocity over time was visualized in parallel with the B-mode scan. After optimization of settings, a video clip of 30 s duration was recorded. Then an appropriately sized blood pressure cuff around the proximal forearm was inflated to 200 mmHg for 5 min. Starting shortly before deflation of the cuff, the recording of a 2 min video of the continuous duplex scan was started. FMD was calculated as maximal relative diastolic diameter increase during post-occlusive reactive hyperaemia as compared to baseline diameter: (diameter_maximal_−diameter_baseline_)/diameter_baseline_*100. FMD was therefore derived from B-mode arterial diameter measurements; Doppler velocity was visualized in parallel to confirm post-occlusive reactive hyperaemia but was not used to calculate the FMD outcome.

### Secondary endpoints

#### Blood pressure, pulse wave velocity, and pulse wave analysis

Peripheral blood pressure, pulse wave velocity, and pulse wave analysis were assessed using the Arteriograph 24 system (Tensiomed, Hungary) and analysed with Tensiowin 24 software. This cuff-based oscillometric device records brachial pressure waveforms from the upper arm and derives PWV, estimated aortic systolic blood pressure, aortic pulse pressure, and aortic augmentation index (AIx) using the manufacturer’s algorithm. Measurements were performed with participants supine after at least 5 min of rest, in duplicate at each time point, and the average was used for analysis.

#### Blood lipid profile

Blood samples were collected before vascular measurements at visits 1 and 2. A 6 mL serum separator vacutainer (Becton Dickinson) was collected from the left arm of all volunteers. The sample was allowed to clot for 30 min before centrifugation at 3 000*×g* for 15 min at room temperature. The resultant serum was aliquoted into six cryovials (Nunc), each containing 500 µL, and then frozen at −80°C for analysis, which was performed using standard methods at Eurofins/Affinity Biolabs.

#### Cardiovascular risk estimation

Ten-year risk of first-onset atherosclerotic cardiovascular disease was calculated using SCORE2/SCORE2-OP, following the 2021 European Society of Cardiology (ESC) cardiovascular prevention guideline.^11^ The algorithm incorporates age, sex, systolic blood pressure, smoking status, and non-high density lipoprotein (HDL) cholesterol, and is calibrated to regional background risk; the United Kingdom is classified as a low-risk region. SCORE2-OP was applied for participants ≥70 years.

#### Adverse event monitoring

Adverse events were monitored throughout the study, with participants instructed to self-report any symptoms or concerns during the home supplementation period.

#### Sample size, randomization, and concealment mechanism

The sample size estimation was based on the primary endpoint FMD and considering the key secondary endpoint PWV. Our previous experiments have shown average FMD values of 6% with a standard deviation of 1%. While the supplement has not been tested in terms of effects on FMD, we expected a change of 1%, which would require studying 17 supplement subjects and 17 control subjects to be able to reject the null hypothesis that the population means of the supplement and control groups are equal with a probability (power) of 0.8. Our previous experiments have shown average PWV values of 9.3 m/s with a standard deviation of 1.3 m/s. To detect a change in PWV of 1 m/s, 28 supplement subjects and 28 control subjects would be needed to reject the null hypothesis that the population means of the supplement and control groups are equal with probability (power) 0.8. The Type I error probability associated with this test of this null hypothesis is 0.05. Taking 5–10% attrition into account, we had planned to recruit a total sample size of 60 (30 supplements and 30 controls). The treatment allocation was assigned based on a computer-generated unrestricted random list of 65 (random.org). Interventions were labelled as A or B, and a sealed envelope was kept by an independent person in a safe space. Concealment was opened after completion of blinded primary endpoint analysis.

### Statistical analyses

All analyses were conducted according to CONSORT recommendations. Continuous variables are presented as mean±SD unless stated otherwise. Primary analyses of changes from baseline to 6 months were analysed using ANCOVA, with group as factor and the corresponding baseline as covariate to adjust for baseline differences and improve precision. We report adjusted between-group differences (ΔΔ), 95% CIs and *P-*values. Supplementary analyses used repeated-measures ANOVA (rmANOVA) to examine outcome trajectories across baseline, 1 h, and 6 months, with time as a within-subject factor and group as a between-subjects factor. For these models, we report main effects of time and group, and the group × time interaction, along with partial eta squared (η^2^p) as an index of effect size. Two-sided *P* < 0.05 was considered significant. Primary analyses were performed in participants with FMD at baseline and 6 months (complete-case, *n* = 43). For secondary outcomes with missing data, analyses were limited to participants with available measurements, no imputation was performed. Correlation analyses used Pearson’s correlations. Analyses were performed in SPSS (IBM, v29).

## Results

### Participants and characteristics

A total of 61 participants were randomized (33 to the intervention and 28 to control), and all received their allocated treatment at visit 1 (*Figure 1*). During follow-up, nine participants in the intervention group and five in the control group did not attend visit 2 for unknown reasons, while three additional participants (control group) discontinued due to self-reported intolerance (mild reflux, *n* = 2) and one unrelated adverse event (leg oedema after travel to a tropical place, suspected parasitic infection). One control group participant was diagnosed with paroxysmal atrial fibrillation and was excluded as no longer eligible. In fact, the participant had an irregular heart rate during a study visit, which triggered a Holter ECG by the GP. Primary analyses were therefore conducted in the 43 participants with complete primary endpoint assessments (primary complete-case analysis: 24 interventions, 19 controls, *Table 3*). Device malfunction (arteriograph) and insufficient sample quality are responsible for missing data in related secondary outcome parameters (*Table 3*).

**Figure 1:**
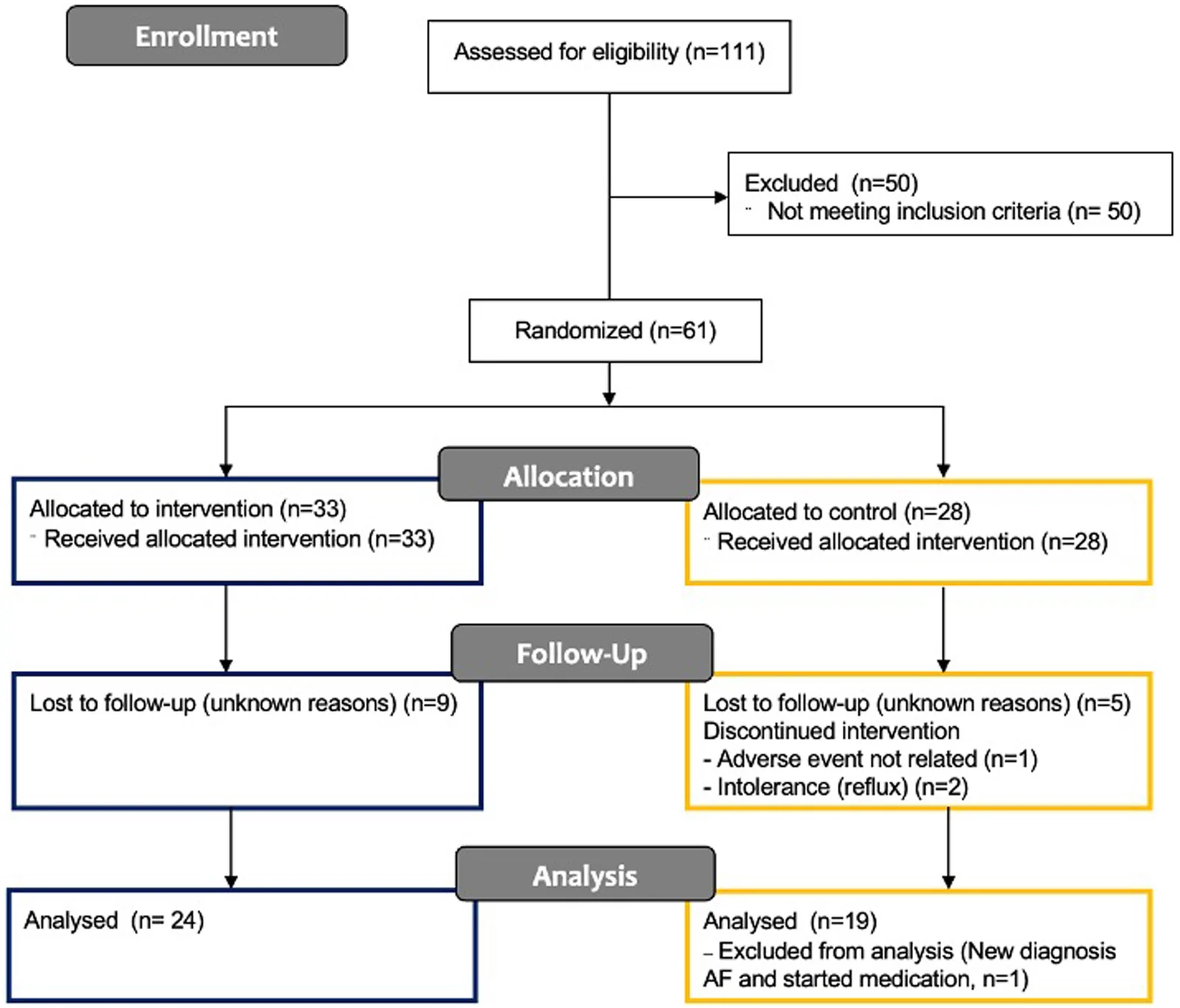
CONSORT diagram.

Baseline characteristics are shown for both the full randomized population and the complete-case population included in the primary analysis (*Table 2*). In the full randomized population, participants allocated to control were older than those allocated to intervention (60 ± 12 vs. 53 ± 8 years, *P* = 0.017). This difference was attenuated in the complete-case analysis population and was no longer statistically significant (59 ± 12 vs. 54 ± 9 years, *P* = 0.146). No statistically significant between-group differences were observed for BMI, glucose, C-reactive protein, lipid profile, peripheral or aortic blood pressure, FMD, PWV, or augmentation index in the analysis population. SCORE2/SCORE2-OP was numerically higher in control participants, consistent with the age difference, but this did not reach statistical significance in either the randomized population (*P* = 0.060) or the complete-case population (*P* = 0.182). Baseline SCORE2/SCORE2-OP values showed significant inverse correlations with baseline FMD (*r* = −0.56, *P* < 0.0001) and correlated with PWV (*r* = 0.32, *P* = 0.043).

**Table 2:** Baseline characteristics of study population randomized to intervention and included in analysis

	Allocated to intervention			Included in analysis		
	Intervention	Control	*P* (*t*-test)	Intervention	Control	*P* (*t*-test)
*n* (m/f)	33 (16/17)	28 (14/14)		24 (12/12)	19 (9/10)	
Age (years)	53 ± 8	60 ± 12	0.017	54 ± 9	59 ± 12	0.146
Height (m)	1.73 ± 0.10	1.70 ± 0.10	0.209	1.73 ± 0.10	1.69 ± 0.09	0.136
Weight (kg)	74 ± 12	70 ± 9	0.140	75 ± 12	69 ± 9	0.104
Body mass index (kg/m^2^)	24.7 ± 3.2	24.2 ± 2.5	0.538	24.8 ± 3.1	24.3 ± 3.1	0.598
Smoker (*n*)	0	0		0	0	
Glucose (mmol/L)	4.9 ± 0.5	4.8 ± 0.4	0.344	4.9 ± 0.5	4.8 ± 0.4	0.361
C-reactive protein (mg/L)	1.7 ± 2.3	2.9 ± 3.5	0.223	1.7 ± 2.3	3.0 ± 3.7	0.182
Total cholesterol (mmol/L)	5.6 ± 1.1	5.6 ± 1.0	0.846	5.6 ± 1.1	5.6 ± 0.9	0.872
LDL cholesterol (mmol/L)	3.4 ± 1.1	3.4 ± 1.0	0.856	3.4 ± 1.1	3.5 ± 1.0	0.655
HDL cholesterol (mmol/L)	1.7 ± 0.3	1.8 ± 0.3	0.784	1.7 ± 0.3	1.8 ± 0.3	0.589
Triglycerides (mmol/L)	1.2 ± 0.7	1.2 ± 0.6	0.860	1.2 ± 0.7	1.1 ± 0.6	0.943
SBP (mmHg)	128.2 ± 13.1	125.4 ± 16.9	0.481	127.9 ± 12.9	124.4 ± 12.9	0.373
DBP (mmHg)	75.1 ± 8.7	74.5 ± 10.5	0.819	74.4 ± 8.9	75.7 ± 9.1	0.645
SCORE-2/SCORE-2OP (10yr%)	3.0 ± 2.3	4.9 ± 4.2	0.060	3.0 ± 2.3	4.2 ± 3.4	0.182
Flow-mediated dilation (%)	5.5 ± 1.9	4.8 ± 2.2	0.211	5.5 ± 1.9	5.0 ± 2.2	0.427
Pulse wave velocity (m/s)	9.0 ± 2.2	8.8 ± 1.9	0.700	9.1 ± 2.4	8.6 ± 2.0	0.490
aoSBP (mmHg)	126.1 ± 16.4	127.1 ± 21.0	0.843	125.0 ± 15.8	127.1 ± 15.7	0.675
aoAix (%)	33.8 ± 15.3	40.3 ± 17.3	0.141	32.5 ± 16.0	40.8 ± 16.6	0.113

LDL, low-density lipoprotein; HDL, high-density lipoprotein; SBP, systolic blood pressure; DBP, diastolic blood pressure; aoSBP, aortic systolic blood pressure; aoAix, aortic augmentation index.

### Primary outcome

FMD increased in the intervention group, whereas values in the control group remained essentially unchanged (*Table 3*; *Figure 2*). At 6 months, the FMD increase was significantly larger in the intervention group (2.6 ± 2.0%) compared with control (−0.1 ± 1.3%), corresponding to a baseline-adjusted ΔΔ6 m of 2.9% (95%CI, 2.1–3.8). In addition, FMD already increased after the first dose by 3.4 ± 2.4% in the intervention group compared with −0.2 ± 0.9% in the control group, resulting in an adjusted between-group difference in change (ΔΔ1 h) of 3.9% (95%CI, 2.7–5.1). Repeated-measures ANOVA indicated a statistically significant overall treatment effect (*P* < 0.0001, Supplementary material online, *Table S1*), with no significant time × treatment group interaction. The chronic change in FMD inversely correlated with baseline FMD (*r* = −0.40, *P* = 0.008) and positively correlated with 1 h FMD changes (*r* = 0.71, *P* < 0.001).

**Figure 2:**
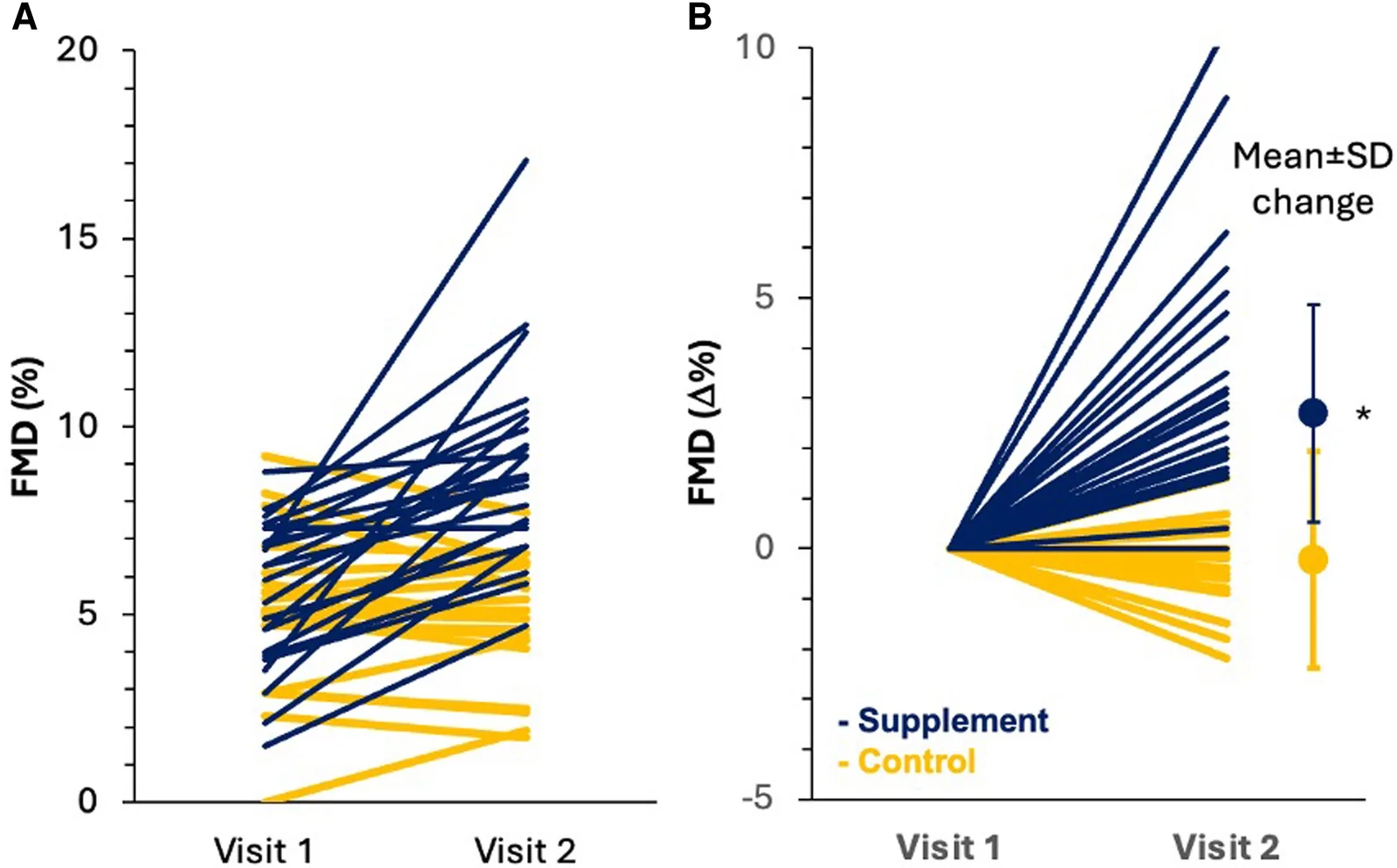
(*A*) Individual flow-mediated dilation (FMD) data in supplement (blue) and control (orange) groups at visit 1 and after chronic daily ingestion of the intervention (visit 2). (*B*) Changes of FMD at visit 2 relative to visit 1, with mean changes in groups. Error bars = SD, * *P* < 0.0001.

**Table 3:** Model-adjusted changes in vascular, haemodynamic and biochemical outcomes at 6 months

Outcome	*n* (Intervention/Control)	Baseline (Intervention vs. Control)	Δ 6 months (Intervention)	Δ 6 months (Control)	ΔΔ 6 months	*P* (group)	η^2^p	Interpretation
		Mean ± SD	Mean ± SD	Mean ± SD	Mean ± SE (95% CI)			
Endothelial function								
FMD (%)	(24/19)	5.5 ± 1.9 vs. 4.8 ± 2.2	+2.6 ± 2.0	−0.1 ± 1.3	**+2.9** **±** **0.4 (2.1–3.8)**	**<0**.**0001**	0.554	Improvement in endothelial function
Arterial stiffness								
PWV (m/s)	(22/18)	9.2 ± 2.4 vs. 8.6 ± 2.0	−0.78 ± 1.39	+0.56 ± 1.40	**−1.18** **±** **0.40 (−2.00 to −0.36)**	**0**.**006**	0.187	Reduction in arterial stiffness
aoSBP (mmHg)	(23/18)	126 ± 16 vs. 127 ± 21	−8.7 ± 7.3	−4.3 ± 11.9	−4.6 ± 3.0 (−10.8 to 1.5)	0.134	0.058	No effect on central blood pressure
aoAix (%)	(23/18)	32.5 ± 16.0 vs. 40.8 ± 16.6	−4.3 ± 7.7	−0.5 ± 15.1	−6.4 ± 3.4 (−13.3 to 0.6)	0.069	0.084	No detectable effect on wave reflection indices
Peripheral blood pressure								
SBP (mmHg)	(24/19)	128 ± 13 vs. 125 ± 17	−8.5 ± 7.0	−1.5 ± 9.6	**−6.1** **±** **2.4 (−10.9 to −1.3)**	**0**.**014**	0.142	Reduction in SBP
DBP (mmHg)	(24/19)	75 ± 9 vs. 74 ± 10	−4.9 ± 5.7	−4.5 ± 7.2	−0.8 ± 1.8 (−4.5 to 2.7)	0.636	0.006	No effect on DBP
Biochemical profile								
Total cholesterol (mmol/L)	(22/17)	5.6 ± 1.1 vs. 5.5 ± 1.0	−0.11 ± 0.61	−0.04 ± 0.75	−0.07 ± 0.21 (−0.50 to 0.36)	0.749	0.003	No effect on total cholesterol
LDL-C (mmol/L)	(22/17)	3.4 ± 1.1 vs. 3.5 ± 1.1	+0.06 ± 0.64	–0.13 ± 0.80	+0.18 ± 0.22 (−0.26 to 0.61)	0.412	0.019	No effect on LDL-C
HDL-C (mmol/L)	(22/17)	1.7 ± 0.3 vs. 1.8 ± 0.3	–0.07 ± 0.15	+0.01 ± 0.33	–0.09 ± 0.07 (−0.24 to 0.06)	0.233	0.039	No effect on HDL-C
Triglycerides (mmol/L)	(19/14)	1.2 ± 0.7 vs. 1.1 ± 0.7	−0.06 ± 0.53	−0.05 ± 0.62	0.03 ± 0.16 (−0.29 to 0.35)	0.871	0.001	No effect on triglycerides
CVD risk								
SCORE2/SCORE2-OP (10 yr %)	(22/17)	3.0 ± 2.3 vs. 4.9 ± 4.2	−0.13 ± 0.39	+0.29 ± 0.82	**−0.45** **±** **0.20 (−0.86 to −0.03)**	**0**.**036**	0.116	Modest SBP-driven reduction in estimated 10-year CVD risk

Δ = change from baseline within group; ΔΔ = between-group difference in change (intervention—control) derived from ANCOVA model adjusted for baseline. Missing values due to technical issues with arteriograph and blood samples are detailed in Methods. Negative ΔΔ values indicate a greater reduction in the intervention group.

### Secondary outcomes

PWV decreased by −0.78 ± 1.39 m/s at 6 months in the intervention group, compared with an increase of +0.56 ± 1.4 m/s in the control group. The resulting ΔΔ6 m was −1.18 m/s (95%CI, −2.0 to −0.36). PWV decreased numerically more in the intervention than in the control group 1 h after the first dose, although the between-group difference at this time point was not statistically significant. Supplementary material online, *Table S1* shows rmANOVA analysis including 1 h and 6-month changes with a statistically significant time × treatment group interaction (*P* = 0.029), indicating a different temporal profile of PWV between groups. Chronic changes in PWV inversely correlated with chronic changes in FMD (*r* = −0.37, *P* = 0.019).

Systolic blood pressure decreased by −8.5 ± 7.0 mmHg at 6 months, whereas the control arm demonstrated a smaller reduction (−1.5 ± 9.6 mmHg). These differences yielded a ΔΔ estimate of −6.1 mmHg (95%CI, −10.9 to −1.3). Similar to FMD, SBP changes were already evident at 1 h after consumption of the first intervention (see Supplementary material online, *Table S1*). Chronic changes in SBP inversely correlated with baseline SBP (*r* = −0.43, *P* = 0.004) and positively with changes in PWV (*r* = 0.40, *P* = 0.011) but not with FMD changes (*r* = −0.17, *P* = 0.284). Diastolic BP changes did not differ significantly between groups.

Central haemodynamic measures followed a similar pattern, with aortic systolic blood pressure decreasing by −8.7 ± 7.3 mmHg in the intervention group and −4.3 ± 11.9 mmHg in the control (between-group difference −4.6 mmHg, 95% CI, −10.8 to 1.5, *P* = 0.134). The rmANOVA analysis showed overall treatment effects (*P* = 0.047). No differences in changes of AIx were observed.

Lipid concentrations, including total cholesterol, low-density lipoprotein (LDL)-C, HDL-C, and triglycerides, did not differ significantly between groups at 6 months.

SCORE2/SCORE2-OP estimates decreased slightly in the intervention group (−0.13 ± 0.39%) and increased in the control group (+0.29 ± 0.82%), yielding an adjusted between-group difference of −0.45% (95% CI, −0.86 to −0.03). Because age, sex, smoking status, and regional calibration were unchanged during follow-up, and non-HDL cholesterol did not differ significantly between groups, the observed difference in SCORE2/SCORE2-OP was driven primarily by the between-group difference in systolic blood pressure.

Finally, we performed a per-protocol sensitivity analysis excluding participants with low adherence based on diaries (<80%, *n* = 2) and outside the defined exposure window (3–6 months, *n* = 2). The results did not materially differ from results of primary complete-case analysis (see Supplementary material online, *Table S2*).

## Discussion

### Summary of principal findings

In this randomized, placebo-controlled study in healthy middle-aged and older adults, daily supplementation with a multi-component nutritional supplement was associated with improvements in vascular function compared with control. The intervention was associated with consistent, sustained increase in flow-mediated dilation and reductions in arterial stiffness and systolic blood pressure, while the lipid profile remained unchanged. These effects were accompanied by a modest reduction in estimated 10-year cardiovascular risk (SCORE2/SCORE2-OP) in this predominantly low-risk population. As SCORE2/SCORE2-OP includes systolic blood pressure and non-HDL cholesterol, but not FMD, PWV or central haemodynamic measures, this reduction was primarily attributable to lowering of systolic blood pressure rather than lipid modification.

### Multi-component formulation and biological rationale

Ageing is increasingly conceptualized as a multifactorial process driven by interconnected molecular and cellular changes that impair physiological integrity, tissue homeostasis, and resilience to age-related diseases,^2^ including cardiovascular disease.^12^ Vascular ageing is one clinically relevant expression of these processes and reflects the cumulative impact of ageing-related changes, including oxidative and mitochondrial stress, impaired stress resilience, chronic low-grade inflammation, cellular senescence, dysregulated nutrient sensing, and extracellular matrix remodelling, on endothelial and vascular smooth muscle cells in both large arteries and the microcirculation.^13^ These changes contribute to endothelial dysfunction, arterial stiffening, microvascular rarefaction, barrier dysfunction, and a pro-atherogenic vascular phenotype, thereby promoting the development and progression of age-related cardiovascular and cerebrovascular disease.^13^ On this basis, interventions that target ageing-related biology have been proposed as a potential strategy to preserve cardiovascular health and delay the development of age-related vascular dysfunction. This study therefore tested whether a geroscience-informed, multi-component nutritional intervention could favourably influence vascular ageing phenotypes in humans.

The formulation evaluated in the present study was selected to combine ingredients with prior evidence of relevance to complementary biological domains implicated in ageing and vascular dysfunction, including endothelial function, redox balance, inflammatory signalling, metabolic regulation, and vascular tone.^14–34^ The rationale was not that any single component would be expected to produce a dominant vascular effect, but that a multi-component nutritional approach may be better aligned with the multifactorial nature of vascular ageing. In support of this formulation-level rationale, the same multi-component formulation has recently been reported to extend lifespan in an obesogenic mouse model, providing preclinical justification for evaluating the combined preparation rather than its individual components.^35^ However, these preclinical findings cannot be directly extrapolated to vascular effects, mechanisms of action, or healthy longevity in humans.

The present study provides preliminary human evidence that this multi-component formulation is associated with improvements in vascular function, arterial stiffness, and systolic blood pressure. However, the study did not include mechanistic endpoints and therefore cannot determine which component(s), combinations of components, or downstream pathways contributed to the observed vascular effects. Further studies incorporating mechanistic biomarkers, component-deconstruction designs, and longer-term clinical outcomes are required to determine whether the observed vascular changes reflect modulation of ageing-related biology and whether they translate into effects on cardiovascular risk or healthy longevity.

### Effects on endothelial function and potential mechanisms

Endothelial function, assessed by brachial artery FMD, improved at the prespecified 6-month endpoint. We also observed an increase in FMD 1 h after the first supervised dose. Several meta-analyses have reported the prognostic value of FMD indicating that an increase in FMD of ∼1% is associated with a ∼10% decrease in CVD risk even in non-CVD populations.^36–38^ Despite participants having baseline FMD in the low–normal range,^39^ the improvement was substantial, underscoring the potential relevance for primary prevention populations. Although the present study did not assess clinical outcomes, the observed FMD improvement supports a favourable shift in endothelial function as an intermediate vascular-risk marker and is directionally consistent with potential cardiovascular risk reduction, particularly when considered alongside the reductions in systolic blood pressure and PWV.

Because of the multi-component composition of the supplement, it is difficult to gain mechanistic insight. Conceptually, the effects of a multi-component nutritional formulation may be mediated by circulating bioactive molecules, including parent compounds, direct metabolites, conjugated metabolites, or gut microbiome-derived catabolites. These molecules may exert short-term effects through receptor-mediated signalling, enzyme modulation, antioxidant or redox effects, altered endothelial nitric oxide bioavailability, changes in vascular smooth muscle tone, or effects on autonomic and haemodynamic regulation. Such mechanisms could plausibly contribute to acute post-dose changes in FMD or blood pressure, although the present study did not measure circulating metabolites, nitric oxide products, autonomic markers, or oxidative-stress endpoints, and therefore cannot assign the acute response to a specific pathway.

With repeated daily exposure, additional mechanisms may become relevant. Recurrent exposure to bioactive molecules and their metabolites may alter endothelial cell phenotype, redox balance, inflammatory tone, mitochondrial function, vascular smooth muscle responsiveness, extracellular matrix turnover, or gut microbiome composition. These changes could produce a 6-month vascular effect that is not simply the sum of repeated acute vasodilator responses. In this context, the 6-month improvement in FMD, PWV, and systolic blood pressure may reflect a combination of residual pharmacodynamic effects from recent dosing and longer-term adaptation of vascular function and/or structure. However, because the final assessment was performed approximately 24 h after the last dose rather than after a formal pharmacokinetic washout, the present study cannot distinguish persistent short-term effects from true structural or cellular vascular adaptation.

Similar patterns of acute and longer-term improvement in FMD have been described for responses to flavanol-rich foods,^4^ citrulline/arginine donors,^40,41^ inorganic nitrate,^42^ and other nutraceuticals,^43^ some of which have been linked to nitric oxide-related pathways.^4^ However, such mechanisms were not directly assessed in the present study.

### Blood pressure and arterial stiffness: implications for vascular ageing

Systolic blood pressure declined meaningfully in the supplement group, with reductions comparable to or larger than those reported for flavan-3-ols, nitrate-rich foods, and lifestyle-based interventions targeting NO pathways.^4,42^ The sustained lowering of both peripheral and central SBP at 6 months suggests an effect that extends beyond transient vasodilation to broader improvements in vascular tone. The magnitude of SBP lowering observed here (6.1 mmHg vs. control) is within the range reported for some lifestyle-based interventions and comparable to effects reported in meta-analyses of antihypertensive drug trials, although such comparisons are indirect and should be interpreted cautiously.^44,45^ The recent ESC guidelines^46^ state that BP reduction with standard doses of any of the five classes of standard antihypertensives (ACE inhibitors, ARBs, dihydropyridine CCBs, diuretics, and beta-blockers) can be expected to be approximately −9/−5 mmHg with office BP and −5/−3 mmHg with ambulatory BP monitoring.^44,45^ A more recent individual patient-level data meta-analysis of 52 large-scale randomized clinical trials in the *Blood Pressure Lowering Treatment Trialists’ Collaboration* reported a difference between active pharmacological treatment and placebo of −5.1/−2.3 mmHg.^44^ While the current study population would not be classified as having ‘arterial hypertension,’ the recent ESC guidelines clearly classify blood pressure of 120–139/70–89 mmHg as elevated and requiring intervention.^46^ Supporting this, an individual participant-level meta-analysis including 344 716 participants reported that a 5 mmHg reduction in systolic BP was associated with an approximately 10% reduction in major cardiovascular events, independent of baseline cardiovascular disease status. This suggests that the observed approximately 6 mmHg SBP reduction could be relevant if sustained long term.^47^

Carotid–femoral pulse wave velocity is a robust index of arterial stiffness, a clinically applicable marker of vascular ageing, and an independent predictor of cardiovascular risk.^48^ The reduction in PWV further supports a favourable change in device-derived arterial stiffness, although the clinical implications in terms of vascular ageing and CV risk require confirmation in larger studies. The magnitude of PWV change (−1.18 m/s vs. control at 6 months) is comparable to or larger than antihypertensives^49^ or other nutraceutical interventions,^7,43,50^ consistent with interventions that reverse components of early vascular ageing.^43^ Improvements may reflect enhanced endothelial-dependent vasodilation, reduced sympathetic tone, or even vascular smooth muscle phenotype or changes in vascular structure. Importantly, central SBP exhibited parallel benefits, underscoring improved aortic function and reduced late systolic load. Together, these findings support further evaluation of the intervention as a potential strategy to attenuate age-associated increases in arterial stiffness.

### Absence of lipid modulation and implications for cardiovascular risk reduction

Lipid concentrations were unchanged, indicating that the modest reduction in estimated 10-year cardiovascular risk was not mediated by lipid lowering. SCORE2/SCORE2-OP incorporates age, sex, smoking status, systolic blood pressure, and non-HDL cholesterol, but does not include FMD, PWV, or central haemodynamic measures. Therefore, because age, sex, and smoking status were unchanged and non-HDL cholesterol did not differ between groups, the observed between-group difference in SCORE2/SCORE2-OP is best interpreted as being driven primarily by the reduction in systolic blood pressure. The concomitant improvements in FMD, PWV, and central haemodynamics provide supportive evidence of favourable changes in vascular phenotype, but they should not be interpreted as direct contributors to the calculated SCORE2/SCORE2-OP reduction. These vascular measures have been associated with cardiovascular risk in previous studies beyond traditional risk factors,^36^ but the present trial did not assess clinical endpoints and was not powered to determine whether changes in these intermediate vascular markers translate into fewer cardiovascular events.

### Limitations

This study had several limitations. Randomization resulted in an age imbalance in the full randomized population, with older participants allocated to control. This imbalance was attenuated among participants included in the complete-case primary analysis and was no longer statistically significant. Nevertheless, age is strongly associated with endothelial function, arterial stiffness, and SCORE2/SCORE2-OP estimates, and residual imbalance may have influenced baseline vascular-risk profiles. The primary analyses adjusted for the corresponding baseline value of each outcome, but the modest sample size limits the extent to which confounding by age or other baseline characteristics can be excluded. Furthermore, this was a single-centre study with a relatively small sample size. Although the sample size was adequate to detect differences in vascular function, it was not powered to allow generalizability to a general population and for clinical events. SCORE2/SCORE2-OP was used as a surrogate risk estimate, and longer-term studies with hard endpoints are required to confirm that vascular effects translate into outcome benefits. Attrition during follow-up reduced analysable numbers, although dropout rates were similar between groups. The study population consisted of generally healthy, non-smoking adults, limiting generalizability to older or higher-risk clinical cohorts. Compliance was monitored by dosing diary only. No objective measure (e.g. pill/sachet count, biochemical marker such as serum ascorbic acid or magnesium levels) was used. Self-reported diaries are well known to overestimate compliance. The multi-component nature of the formulation is an important limitation for mechanistic interpretation. The study cannot identify the active ingredient(s), determine whether additive or synergistic interactions occurred, define dose-response relationships, or establish specific molecular pathways. Mechanistic explanations should therefore be considered hypothesis-generating. The 6-month endpoint was assessed approximately 24 h after the last dose, which avoided immediate post-ingestion measurement but did not constitute a formal pharmacokinetic washout; residual pharmacodynamic effects from recent dosing therefore cannot be excluded.

## Conclusion

Daily supplementation with a multi-component nutritional formulation was associated with sustained improvements in endothelial function, arterial stiffness, and systolic blood pressure, and was accompanied by a modest reduction in estimated 10-year cardiovascular risk driven primarily by lower systolic blood pressure rather than lipid change. These preliminary findings support further evaluation of multi-component nutritional strategies for maintaining vascular health during mid-life, including studies with mechanistic endpoints and longer-term clinical outcomes.

## Supplementary Material

oeag157_Supplementary_Data

## Data Availability

The current study data are not publicly available but may be available from the corresponding author on reasonable request and with appropriate approvals.
