# Supplementary material for: Acute and 6-month vascular effects of a multi-component nutritional supplement: a randomized controlled trial
Source: Eur Heart J Open. 2026 Nov 4;6(6):oeag157. doi: 10.1093/ehjopen/oeag157 (PMC13730552; doi:10.1093/ehjopen/oeag157)
Supplement: Supplementary file 1 [file oeag157_supplementary_data.docx]

**Supplemental data**

**Supplementary Table 1:** Model-adjusted changes in vascular, haemodynamic and biochemical outcomes at 1 hour and 6 months. Δ = change from baseline within group; ΔΔ = between-group difference in change (intervention – placebo) derived from rmANOVA model adjusted for baseline. Missing values due to technical issues with Arteriograph and blood samples are detailed in Methods. Negative ΔΔ values indicate a greater reduction in the intervention group.

**Supplementary Table 2: Per Protocol sensitivity analysis excluding participants with low adherence (<80%, n=2) and outside exposure window (3-6 months, n=2).** Model-adjusted changes in vascular, haemodynamic and biochemical outcomes at 6 months. Δ = change from baseline within group; ΔΔ = between-group difference in change (intervention – control) derived from ANCOVA model adjusted for baseline. Missing values due to technical issues with Arteriograph and blood samples are detailed in Methods. Negative ΔΔ values indicate a greater reduction in the intervention group.
